# Comparative Abilities of Body Mass Index, Waist Circumference, Abdominal Volume Index, Body Adiposity Index, and Conicity Index as Predictive Screening Tools for Metabolic Syndrome among Apparently Healthy Ghanaian Adults

**DOI:** 10.1155/2019/8143179

**Published:** 2019-09-02

**Authors:** Lawrence Quaye, William Kwame Boakye Ansah Owiredu, Nafiu Amidu, Peter Paul Mwinsanga Dapare, Yussif Adams

**Affiliations:** ^1^Department of Biomedical Laboratory Science, University for Development Studies, Tamale, Ghana; ^2^Department of Molecular Medicine, Kwame Nkrumah University of Science and Technology, Kumasi, Ghana

## Abstract

The prevalence of the metabolic syndrome (MetS) continues to increase. There is therefore the need for early detection to avert possible adverse outcomes. Several anthropometric methods have been suggested to predict MetS, but no consensus has been reached on which is best. The aim of the study was to explore the comparative abilities of conicity index, body adiposity index, abdominal volume index, body mass index, and waist circumference in predicting cardiometabolic risk among apparently healthy adults in the Tamale metropolis. This study was a cross sectional study conducted from September 2017 to January 2018, among one hundred sixty (160) apparently healthy normoglycemic normotensive adults. A self-designed questionnaire was administered to gather sociodemographic data. Anthropometric and haemodynamic measurements were also taken. Blood samples were collected for fasting blood glucose (FBG) and lipid profile. MetS was classified using the harmonised criteria as indicated by the joint interim statement (JIS). Of 160 participants, 42.5% were male and 57.5% were female. Body mass index (BMI) and waist circumference (WC) associated better with MetS and other cardiovascular risk factors. Generally, BMI and WC showed largest area under curves (AUCs) than abdominal volume index (AVI), body adiposity index (BAI), and conicity index (CI) in predicting MetS and its components. Upon gender stratification, AVI and CI had the larger AUCs in females whiles BMI remained the superior index in males. Whiles BMI and WC remained useful parameters, they were not useful in predicting MetS and its components in the female population in this study.

## 1. Introduction

Obesity has been well known as a potential cardiometabolic risk factor for some years. It is foreseen that by 2030 up to 57.8% of adults worldwide would become overweight or obese [1]. Body mass index (BMI) is a method that is broadly used to categorise general body weight [2]. BMI is very useful but notwithstanding its widespread use; it is just a substitute measurement of body fat, does not offer a true indication of body composition [3], and is sometimes affected by age, gender, and ethnic differences [4]. To measure central obesity, a variety of indices have been proposed with the most common being waist circumference (WC) and waist-to-hip ratio (WHR). Body adiposity index (BAI), abdominal volume index (AVI), and conicity index (CI) have also been proposed [5–7]. The body adiposity index (BAI) was proposed as an index to assess adiposity, to cater for the shortcomings of BMI. It can be computed from the height and hip circumference, and it can be useful in indicating body fat percentage (BF%) in grown-ups [6]. Another index of abdominal adiposity is the conicity index (CI). This has a hypothetical range, includes an integral modification of waist circumferences for weight and height, and excludes the hip circumference to measure fat distribution [6]. The abdominal volume index (AVI) is an anthropometric tool for measuring general volume. It is seen to have a very close link with the impairment of glucose metabolism [8].

There are propositions that the cardiometabolic problems of obesity are less associated with overall adiposity than visceral adiposity [9]. Therefore, alternative measures of adiposity that reflect body fat distribution, such as waist-to-hip ratio (WHR) and waist circumference (WC), were developed. Waist circumference was suggested to be the most preferred within these measures because it has exceptional connection with superior techniques like abdominal fat imaging and greater linkage with CVD, particularly diabetes [5, 9, 10]. WC does not, however, include the variations in height, hence, possibly underassessing and overassessing risk for short and tall individuals, respectively [11]. Subsequently, some scientists proposed other measures like AVI, BAI, and CI [6, 8].

A complete agreement has not been reached about the best indices for assessing the status and risk of MetS. The best obesity measure to use as a predictor of cardiometabolic risk remains elusive in sub-Saharan Africa, particularly in a Ghanaian population of apparently healthy adults. Thus, additional research is necessary in populations and ethnic groups where the varieties of anthropometric measures especially the newly proposed AVI, BAI, and CI have not been expansively analysed and studied. The aim of this study was therefore to assess the comparative abilities of anthropometric indices of adiposity and obesity (body adiposity index (BAI), abdominal volume index (AVI), and conicity index (CI)) in predicting metabolic syndrome (Metabolic score) among apparently healthy adults.

## 2. Methods

### 2.1. Study Population

This study was a cross-sectional study conducted among apparently healthy adults (20–80 years) with no history of diabetes or hypertension within the Tamale metropolis, from September 2017 to January 2018.

#### 2.1.1. Exclusion Criteria

Diabetics, hypertensives, persons undergoing treatment for diabetes or hypertension, persons with a fasting blood glucose >7.0 mmol/l or HbA1c ≥ 6.5% at the time of the study, pregnant women, persons showing signs of any acute illnesses and persons with other chronic diseases, i.e., cancer, were excluded from this study. Subjects were excluded based on their responses to questions regarding the exclusion criteria, physical assessment, blood glucose, glycated haemoglobin results, and blood pressure readings.

#### 2.1.2. Sample Size

The minimum sample size for the study was calculated to be 105 adults, based on the assumption that 7.4% of the normal adult population has metabolic syndrome [12], with an expected difference of 5% between the sample and the general population and a type I error (*α*) of 0.05.

In this study, which was limited to only apparently healthy adults who answered at least 75% of the questions in the questionnaire and did not have an FBG of >7.0 mmol/l or an HbA1c of >6.5, the sample size was recalculated to adjust for any possible loss of respondents. Assuming a response rate of 90%, the sample size is recalculated as 105/0.90. Using the preceding formula, the calculated sample size was approximately 117. One hundred twenty (120) participants were therefore targeted for this study.

### 2.2. Data Collection

#### 2.2.1. Sociodemographic and Anthropometric Data

A self-designed semistructured questionnaire was administered to consented study participant for sociodemographic data. The questionnaire was used to capture sociodemographic variables such as age and gender, among others. Weight, to the nearest 0.1 kg in light clothing, was measured using a digital flat floor weighing scale (with weighing capacity of 250 kg) manufactured by SECA (Hamburg, Germany), and height to the nearest 1 cm was measured using a portable microtoise (measuring range: 0 cm to 220 cm) manufactured by SECA. Waist circumference (to the nearest centimetre) was measured with a Gulick II spring-loaded measuring tape (Gay Mill, WI) midway between the inferior angle of the ribs and the suprailiac crest. Hip circumference was measured as the maximal circumference over the buttocks in centimetre.

#### 2.2.2. Anthropometric Calculations

BMI was calculated as body weight in kg/height in m^2^; WHR was calculated as waist circumference (cm) divided by hip circumference (cm); BAI was calculated as proposed by Bergman et al., [6]:

(1) $$\begin{array}{c} \text{AVI}=\frac{\left[2\text{ cm}\times {\left(\text{waist}\left(\text{cm}\right)\right)}^{2}+0.7\text{ cm}\times {\left(\text{waist }\left(\text{cm}\right)-\text{hip}\left(\text{cm}\right)\right)}^{2}\right]}{1000},\\ \text{BAI}=\frac{\text{hip circumference}\left(\text{cm}\right)}{\text{height}{\left(\text{m}\right)}^{1\text{.}5}}-18,\\ \text{CI}=\frac{\text{waist circumference}\left(\text{m}\right)}{0.109\sqrt{\text{weight}\left(\text{kg}\right)/\text{height}\left(\text{m}\right)}}\;. \end{array}$$

#### 2.2.3. Blood Pressure

Blood pressure was measured in sitting position at the level of the heart, with a sphygmomanometer cuff and a stethoscope. Measurements were taken from the left brachial artery after subjects had been sitting for at least five (5) minutes in accordance with the recommendation of the American Heart Association [13]. The fifth Korotkoff sound, phase V (absence of sound) instead of phase IV Korotkoff sound (muffling), was used for the determination of the diastolic value. Triplicate measurements were taken with a five- (5-) minute rest interval between measurements, and the mean value was recorded to the nearest 2.0 mm·Hg.

#### 2.2.4. Sample Collection, Preparation, and Analysis

Ten milliliters (10 ml) of venous blood sample was collected under strict aseptic conditions from each participant in the morning between 07.00 to 09.00 GMT into fluoride oxalate tube, serum separator tubes (SSTs), and ethylenediamine tetraacetic acid (EDTA) anticoagulated tube (Becton Dickinson, Rutherford, NJ), after an overnight fast. Samples in the fluoride oxalate tubes were used for fasting blood glucose measurement whilst samples in the evacuated gel tubes were centrifuged at 3000 g for 5 minutes and the serum was aliquoted and stored in cryovials at a temperature of −80°C until time for biochemical assays. Lipid profile and fasting blood glucose levels were determined using the Mindray BS-240 Chemistry Analyser (Mindray, China); MedSource Diagnostics reagents were used in all of these assays.

### 2.3. Definitions of Terms

#### 2.3.1. Metabolic Syndrome: Harmonised Criteria in the Joint Interim Statement (JIS)

Metabolic syndrome is defined according to the criteria Joint Interim Statement (JIS) to include individuals with any three or more of the following five components: (1) abdominal obesity (waist circumference, male ≥ 94, female ≥ 80); (2) high TG ≥ 1.7 mmol/L (150 mg/dl); (3) low HDL-C: male < 1.0, female < 1.3 mmol/L (4) high BP (systolic BP ≥ 130 mm·Hg or diastolic BP ≥ 85 mm·Hg or treatment of hypertension); (5) high fasting glucose ≥ 5.6 mmol/l [14].

### 2.4. Statistical Analysis

All analyses were performed using MedCalc® version 10.2.0.0 (http://www.medcalc.be) for windows and GraphPad version 6.0, San Diego California, USA. MetS stratifications, associations, and receiver operator characteristics (ROCs) were assessed using the Harmonised (JIS) criteria. The data were presented as mean ± SD or percentages. The unpaired *T*-test was used to compare continuous variables. Association between variables was assessed with linear regression analysis. Receiver operator characteristics (ROC) was used to compare the relative abilities of various parameters to predict MetS and other cardiovascular risk factors. In all cases, a *p* value < 0.05 was considered significant.

## 3. Results

### 3.1. Anthropometry of Studied Population

Out of the 160 subjects studied, 68 (42.5%) were males and 92 (57.5%) females. The average age of the studied population was 42.8 ± 14.5 years. Respondents with MetS were significantly older (48.2 ± 12.9 years; *p*=0.030) than their counterparts without MetS (41.6 ± 14.6 years). The average BMI, WC, CI, AVI, and BAI were 25.2 ± 5.1 kg/m^2^, 84.4 ± 15.3 cm, 1.2 ± 0.16 m^3/2^·kg^−1/2^, 15 ± 6.9, and 28.1 ± 7.1%, respectively, with respondents of MetS recording significantly higher BMI (*p* < 0.001), WC (*p* < 0.001), AVI (*p*=0.002), and BAI (*p* < 0.001) than their counterparts (Table 1).

### 3.2. Association between CI, AVI, BAI, WC, BMI, and Cardiometabolic Risk Factors

Linear regression analysis was used to assess the association between various anthropometric parameters and selected cardiometabolic risk factors. As shown in Table 2, CI, AVI, BAI, WC, and BMI significantly associated with total cholesterol and LDL-c. AVI, WC, and BMI were significantly associated with triglyceride and VLDL-c. BAI and BMI had a direct relation with HDL-c and SBP, whiles BAI, WC, and BMI related directly with DBP. Only BMI however was associated with FBG. All parameters showed significant associations with MetS score; however, BMI and WC showed stronger associations than AVI, BAI, and CI as shown in Table 2.

WC (abdominal obesity) and BMI (overall obesity) generally showed more associations with the cardiometabolic risk factors, with slight variations in the strengths and number of associations between the two as shown in Table 2.

### 3.3. Receiver Operator Characteristics (ROC) for CI, AVI, BAI, WC, and BMI in the Studied Population

**Figure 1:**
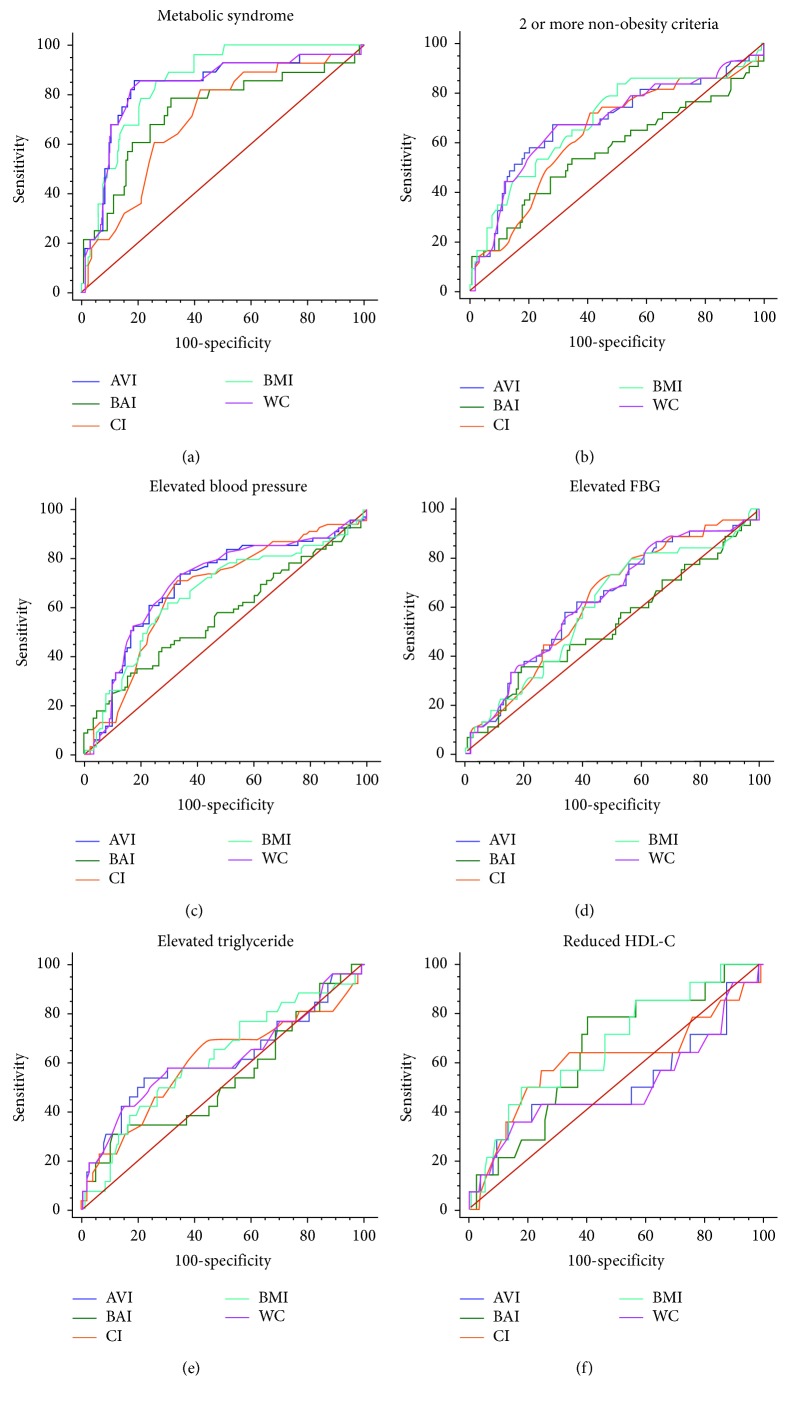
ROC curves for MetS. The relative abilities of AVI, BAI, CI, BMI, and WC are compared to identify respondents with MetS and its components.

The ROC curves and the area under curve (AUC) comparing the predictive abilities of CI, AVI, BAI, WC, and BMI for MetS and its individual components are shown in Figure 1 and Table 3. BMI had the largest AUC for MetS (0.85 (0.79–0.91)); both BMI and WC showed similar AUCs in predicting the presence of a cluster of 2 or more non-WC components for MetS, with both recording similar AUCs and AVI being the second largest in both cases. The ability to predict the presence of an elevated BP and elevated FBG was better done by both AVI and WC, with both showing similar AUCs in all situations while BMI had a larger AUC for the prediction of the presence of elevated triglyceride and reduced HDL-c with AVI and WC being the second largest in both situations, with similar AUCs as shown in Table 3.

### 3.4. Receiver Operator Characteristics (ROC) for CI, AVI, BAI, WC, and BMI among Male Respondents

**Figure 2:**
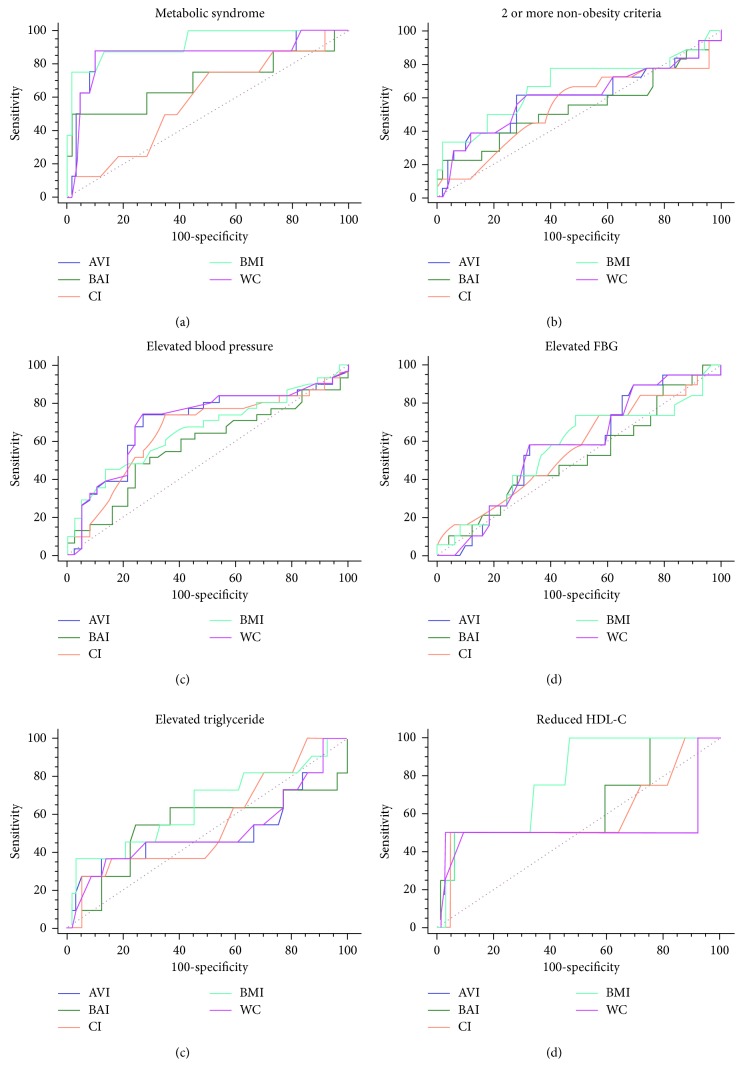
ROC curves for MetS in male participants. The relative abilities of AVI, BAI, CI, BMI, and WC are compared to identify respondents with MetS and its components.

The ROC curves and the area under curve (AUC) comparing the predictive abilities of CI, AVI, BAI, WC, and BMI for MetS and its individual components in male respondents are shown in Figure 2 and Table 4. BMI had the largest AUC for MetS (0.93 (0.84–0.98)), a cluster of 2 or more non-WC components for MetS (0.68 (0.55–0.79)), elevated triglyceride (0.64 (0.52–0.75)), and reduced HDL-c (0.78 (0.66–0.87)), with AVI and WC being the second largest in all situations, with similar AUCs. The ability to predict the presence of an elevated BP and elevated FBG were better done by both AVI and WC, with both showing similar AUCs in both situations as shown in Table 4.

### 3.5. Receiver Operator Characteristics (ROC) for CI, AVI, BAI, WC, and BMI among Female Respondents

**Figure 3:**
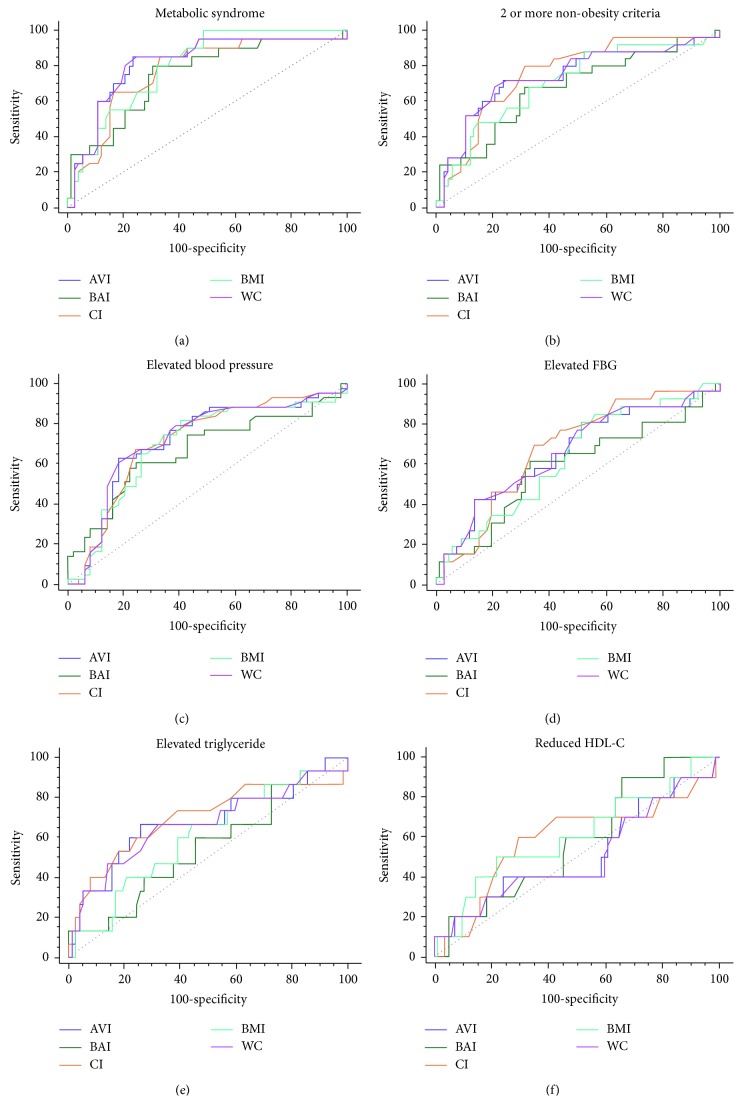
ROC curves for MetS in female participants. The relative abilities of AVI, BAI, CI, BMI, and WC are compared to identify respondents with MetS and its components.

The ROC curves and the area under curve (AUC) comparing the predictive abilities of CI, AVI, BAI, WC, and BMI for MetS and its individual components in female respondents are shown in Figure 3 and Table 5. AVI and WC had the largest AUCs for MetS (0.81 (0.72–0.89) for both); CI showed the largest AUC in predicting the presence of a cluster of 2 or more non-WC components for MetS. The ability to predict the presence of an elevated BP and elevated FBG was better done by WC and CI, respectively. CI had a larger AUC for the prediction of the presence of elevated triglyceride whilst reduced HDL-c was better predicted by BMI as shown in Table 5.

## 4. Discussion

New methods including the AVI, BAI, and CI have been proposed to make up for the observed shortcomings of the older methods. This study therefore assessed the comparative abilities of these methods in predicting MetS among apparently healthy adults.

In the present study, the older methods WC and BMI were found to be more strongly associated with the risk factors than the newer methods. BMI and WC showed more associations with the cardiovascular risk factors, and in cases where AVI, BAI, and CI also showed associations, the association was stronger in BMI and WC. Similar results have also been reported by Lam et al. [15], Shidfar et al. [16], and Bennasar-Veny et al. [17]. Furthermore, BMI and WC showed better predictability for the MetS and its components in the general population. Similar findings have also been reported by several studies [18–21].

Upon stratification, however, a better performance of CI and AVI among a female population as seen in this study has also been reported by Motamed et al. [22] and Wang et al. [23]. These findings further highlight the effect of the waist and hip measures on the variations in the comparative abilities of the indices to predict MetS and its components. The differences in the relative performances of these indices between the male and female population are attributable to the differences in waist and hip circumferences and the resultant effect of the differences in the general body fat distribution. Furthermore, though height is applied in the CI formula, the influence of a higher height of men will be reduced by a higher weight; hence, its inability to properly predict cardiometabolic outcomes in men. Also, in the AVI formula when WC < HC an increase in HC leads to an increase in AVI [22]. Since females have a larger HC than WC, increase in HC values commonly leads to increase in AVI values, hence the variations in the predictive abilities in the two genders.

The arguments about whether abdominal or overall obesity better associates with the cardiometabolic risk continue with no consensus. This study also sought to assess the comparative associations of abdominal obesity indices and overall obesity indices with cardiometabolic risk. From this study, there was no clear superior parameter for the prediction of MetS as far as abdominal and overall obesity are concerned. The two main measures of abdominal and overall obesity, i.e., WC and BMI, showed similar associations with cardiometabolic risk factors and the MetS score. This is in agreement with findings by Vazquez et al. [24]. The finding in this study is perhaps due to the fact that not only visceral adiposity as measured by WC but also subcutaneous adiposity as measured by BMI is associated with the release of inflammatory markers [25]. Subcutaneous adipose tissues as well as visceral adipose tissues have been shown to be positively and similarly correlated with circulating inflammatory adipokines, CRP, fibrinogen, and IL-6 [25]. In the study by Pou et al. [25], subcutaneous adipose tissue was found to be more strongly associated with fibrinogen and visceral adipose tissue was found to be more strongly associated with CRP and IL-6, all of which have been implicated in the pathogenesis of the MetS and its adverse outcomes.

Furthermore, the misclassification error of anthropometric measurements in characterising the body fat stores could also be the reason for the unclear superiority of any of the two measures. WC is considered to be a surrogate marker of visceral adiposity [26], and because visceral fat is believed to be more metabolically active than other fat depots such as subcutaneous fat [27], abdominal adiposity measures such as WC are expected to be more strongly associated with metabolic abnormalities and cardiovascular disease risk than is BMI since BMI is a measure of general adiposity. However, in the larger context of visceral and subcutaneous adipose tissue, WC actually estimates both VAT and abdominal SAT, with studies finding WC to actually be more highly correlated to SAT than to VAT [28].

It is worth noting that, in the female population however, abdominal obesity as indicated by WC and its derivative CI showed superior abilities in predicting MetS and its components than the overall obesity as indicated by the BMI. In the normal distribution of adipose tissues in men and women, women tend to build up excess fat in the lower body gluteal area (i.e., thighs and buttocks), while in future, they tend to accrue excess fat in the upper body area (i.e., central obesity) [29]. Furthermore, women naturally store fat subcutaneously, and men store fat viscerally. A justification for the higher cutoff of ≥94 cm for abdominal obesity in men as compared to the ≥80 cm for females is as shown in the definitions for MetS. This means that in a population of normal adults where male and female are showing similar average measures for WC, WC and its derivatives, i.e., CI will be more correlated to adverse cardiovascular outcomes in females than in males.

The generalisability of the current findings may be problematic due to the relatively smaller sample size. The cross-sectional design does not allow for assessment of the direction of risk factors and health outcomes. From this design, it is indeterminable if obesity or elevated fasting blood glucose occurred before or after risk factors developed.

## 5. Conclusion

This study highlights the usefulness of WC and BMI compared to newer methods, i.e., AVI, BAI, and CI, but BMI may not be useful in a female population as this study shows better performance by AVI and CI in the prediction of MetS and its components in females. Furthermore, in the general population, both abdominal obesities as measured by WC and overall obesity as measured by BMI show similar associations with the cardiometabolic risk factors, whiles their performances differ in females.

## Figures and Tables

**Table 1:** Anthropometric characteristics of studied population stratified by MetS.

Variables	Total (*n* = 160)	No MetS (*n* = 132)	MetS (*n* = 28)	*p* value
Age (years)	42.8 ± 14.5	41.6 ± 14.6	48.2 ± 12.9	0.030
Weight (kg)	68.7 ± 13.6	66.2 ± 12.0	80.6 ± 14.2	<0.001
Height (m)	1.7 ± 0.1	1.7 ± 0.1	1.6 ± 0.1	0.126
BMI (kg/m^2^)	25.2 ± 5.1	24.1 ± 4.4	30.3 ± 5.1	<0.001
WC (cm)	84.4 ± 15.3	82.1 ± 14.9	95.5 ± 11.9	<0.001
HC (cm)	97.4 ± 12.1	95.7 ± 10.9	105.4 ± 14.1	<0.001
WHR (cm)	0.87 ± 0.17	0.87 ± 0.18	0.91 ± 0.10	0.168
CI (m^3/2^·kg^−1/2^)	1.20 ± 0.16	1.19 ± 0.17	1.25 ± 0.11	0.106
AVI	15.0 ± 6.9	14.2 ± 7.1	18.7 ± 4.4	0.002
BAI (%)	28.1 ± 7.1	27.1 ± 6.5	32.9 ± 8.0	<0.001

BMI: body mass index, WC: waist circumference, HC: hip circumference, WHR: waist to hip ratio, CI: conicity index, AVI: abdominal volume index, BAI: body adiposity index. Continuous data are presented as mean ± SD and compared using *T*-test.

**Table 2:** Linear regression analysis selected anthropometric parameters and indicators of cardiometabolic risk.

Variable	CI		AVI		BAI		WC		BMI	
	*β*	*r* ^2^	*β*	*r* ^2^	*β*	*r* ^2^	*β*	*r* ^2^	*β*	*r* ^2^
SBP (mmHg)	−0.57	0.00	−0.05	0.00	0.55^*∗∗*^	0.06	0.08	0.01	0.51^*∗*^	0.03
DBP (mmHg)	11.45	0.02	0.23	0.02	0.40^*∗∗*^	0.05	0.16^*∗*^	0.04	0.38^*∗*^	0.02
FBG (mmol/L)	0.25	0.00	0.01	0.00	0.00	0.00	0.01	0.02	0.03^*∗*^	0.03
Total cholesterol (mmol/L)	1.95^*∗*^	0.05	0.06^*∗∗∗*^	0.08	0.04^*∗∗*^	0.04	0.03^*∗∗∗*^	0.10	0.09^*∗∗∗*^	0.11
Triglyceride (mmol/L)	0.76	0.02	0.02^*∗*^	0.04	−0.01	0.01	0.01^*∗∗*^	0.05	0.03^*∗*^	0.04
HDL-c (mmol/L)	0.80	0.02	0.02	0.01	0.02^*∗*^	0.03	0.01	0.02	0.03	0.03
LDL-c (mmol/L)	0.80^*∗*^	0.03	0.03^*∗*^	0.06	0.02^*∗∗*^	0.04	0.01^*∗∗*^	0.07	0.04^*∗∗*^	0.07
VLDL-c (mmol/L)	0.35	0.02	0.01^*∗*^	0.04	−0.01	0.01	0.01^*∗∗*^	0.05	0.01^*∗*^	0.04
MetS score	1.95^*∗∗*^	0.08	0.07^*∗∗∗*^	0.15	0.06^*∗∗∗*^	0.11	0.04^*∗∗∗*^	0.23	0.12^*∗∗∗*^	0.29

^*∗*^Regression is significant at the 0.05 level. ^*∗∗*^Regression is significant at the 0.01 level. ^*∗∗∗*^Regression is significant at the 0.001 level.

**Table 3:** AUC for AVI, BAI, CI, BMI, and WC to identify respondents with MetS and its components.

Variable	AVI	BAI	CI	BMI	WC
MetS	0.83 (0.76–0.89)	0.74 (0.66–0.80)	0.70 (0.63–0.77)	0.85 (0.79–0.91)	0.83 (0.76–0.89)
2 or more non-obesity criteria	0.68 (0.61–0.76)	0.57 (0.49–0.64)	0.64 (0.56–0.71)	0.69 (0.61–0.76)	0.69 (0.61–0.76)
Elevated BP	0.70 (0.62–0.77)	0.57 (0.49–0.65)	0.67 (0.59–0.74)	0.66 (0.58–0.73)	0.70 (0.63–0.77)
Elevated FBG	0.63 (0.55–0.70)	0.53 (0.45–0.61)	0.62 (0.54–0.70)	0.60 (0.52–0.67)	0.63 (0.55–0.70)
Elevated triglyceride	0.61 (0.53–0.69)	0.53 (0.45–0.61)	0.60 (0.52–0.68)	0.62 (0.54–0.69)	0.61 (0.53–0.69)
Reduced HDL-c	0.51 (0.43–0.59)	0.65 (0.57–0.72)	0.61 (0.53–0.68)	0.67 (0.59–0.74)	0.50 (0.42–0.58)

Results are expressed as the area under curve (confidence interval).

**Table 4:** AUC for AVI, BAI, CI, BMI, and WC to identify male respondents with MetS and its components.

Variable	AVI	BAI	CI	BMI	WC
MetS	0.85 (0.75–0.93)	0.69 (0.57–0.80)	0.58 (0.46–0.70)	0.93 (0.84–0.98)	0.85 (0.74–0.93)
2 or more non-obesity criteria	0.60 (0.48–0.72)	0.54 (0.41–0.66)	0.55 (0.42–0.67)	0.68 (0.55–0.79)	0.61 (0.48–0.72)
Elevated BP	0.70 (0.57–0.80)	0.58 (0.45–0.70)	0.65 (0.53–0.77)	0.66 (0.53–0.77)	0.70 (0.58–0.81)
Elevated FBG	0.57 (0.45–0.69)	0.52 (0.39–0.64)	0.56 (0.43–0.68)	0.56 (0.44–0.68)	0.57 (0.45–0.69)
Elevated triglyceride	0.51 (0.39–0.64)	0.54 (0.41–0.66)	0.55 (0.42–0.67)	0.64 (0.52–0.75)	0.51 (0.39–0.64)
Reduced HDL-c	0.53 (0.40–0.65)	0.65 (0.52–0.76)	0.6 (0.47–0.71)	0.78 (0.66–0.87)	0.52 (0.39–0.64)

Results are expressed as area under curve (confidence interval).

**Table 5:** AUC for AVI, BAI, CI, BMI, and WC to identify female respondents with MetS and its components.

Variable	AVI	BAI	CI	BMI	WC
MetS	0.81 (0.72–0.89)	0.75 (0.65–0.83)	0.77 (0.67–0.85)	0.80 (0.70–0.88)	0.81 (0.72–0.89)
2 or more non-obesity criteria	0.74 (0.64–0.83)	0.67 (0.57–0.77)	0.76 (0.65–0.84)	0.70 (0.60–0.80)	0.74 (0.64–0.83)
Elevated BP	0.71 (0.61–0.80)	0.66 (0.56–0.76)	0.71 (0.61–0.80)	0.70 (0.59–0.79)	0.72 (0.62–0.81)
Elevated FBG	0.65 (0.55–0.75)	0.58 (0.48–0.69)	0.68 (0.57–0.77)	0.63 (0.52–0.73)	0.66 (0.55–0.76)
Elevated triglyceride	0.67 (0.57–0.77)	0.55 (0.44–0.65)	0.69 (0.59–0.79)	0.60 (0.49–0.70)	0.67 (0.56–0.76)
Reduced HDL-c	0.51 (0.57–0.77)	0.58 (0.44–0.65)	0.59 (0.59–0.79)	0.61 (0.49–0.70)	0.51 (0.56–0.76)

Results are expressed as area under curve (confidence interval).

## Data Availability

The data are part of bigger composite data from a project. The data will however be extracted and provided if required.
